# Molecular diagnosis of genodermatoses in india

**DOI:** 10.1186/1755-8166-7-S1-I18

**Published:** 2014-01-21

**Authors:** Parag Tamhankar

**Affiliations:** 1 ICMR-Genetic Research Centre, National Institute of Research in Reproductive Health [NIRRH] Jehangir Merwanji Street, Parel, Mumbai, India

## 

Genodermatoses refer to inherited diseases of skin structure and function. Several genodermatoses present with multisystem involvement lead to increased morbidity and mortality. Genetic Research Centre focused on identifying molecular basis of such dreadful skin diseases with recessive inheritance. This would help us identify common mutations, founder effects etc that would reduce the cost of screening patients and their carrier parents. During the years 2011-13, 100 patients were referred to the centre with genodermatoses. The commonest group was ichthyosis followed by epidermolysis bullosa, ectodermal dysplasia, albinism, cutis laxa, progeroid conditions, precancerous conditions xeroderma pigmentosum, Rothmund Thomson syndrome, dyskeratosis congenita. Genetic heterogeneity is very common and molecular diagnosis requires an extensive effort. Recurrent mutations in unrelated families were seen in families with xeroderma, Griscelli. Prenatal diagnosis could be provided for ichthyosis, infantile hyalinosis and progeria. This is the largest cohort of mutation proven patients with genodermatoses from India.

